# VisualTE: a graphical interface for transposable element analysis at the genomic scale

**DOI:** 10.1186/s12864-015-1351-5

**Published:** 2015-02-27

**Authors:** Sébastien Tempel, Emmanuel Talla

**Affiliations:** Aix Marseille Université, CNRS, LCB UMR 7283, Marseille, 13402 France

**Keywords:** VisualTE, Transposable element, Graphical interface

## Abstract

**Background:**

Transposable elements are mobile DNA repeat sequences, known to have high impact on genes, genome structure and evolution. This has stimulated broad interest in the detailed biological studies of transposable elements. Hence, we have developed an easy-to-use tool for the comparative analysis of the structural organization and functional relationships of transposable elements, to help understand their functional role in genomes.

**Results:**

We named our new software VisualTE and describe it here. VisualTE is a JAVA stand-alone graphical interface that allows users to visualize and analyze all occurrences of transposable element families in annotated genomes. VisualTE reads and extracts transposable elements and genomic information from annotation and repeat data. Result analyses are displayed in several graphical panels that include location and distribution on the chromosome, the occurrence of transposable elements in the genome, their size distribution, and neighboring genes’ features and ontologies. With these hallmarks, VisualTE provides a convenient tool for studying transposable element copies and their functional relationships with genes, at the whole-genome scale, and in diverse organisms.

**Conclusions:**

VisualTE graphical interface makes possible comparative analyses of transposable elements in any annotated sequence as well as structural organization and functional relationships between transposable elements and other genetic object. This tool is freely available at: http://lcb.cnrs-mrs.fr/spip.php?article867.

**Electronic supplementary material:**

The online version of this article (doi:10.1186/s12864-015-1351-5) contains supplementary material, which is available to authorized users.

## Background

Transposable elements (TEs) are repeated DNA sequences that can represent a large fraction of the genomic DNA in eukaryotic species [1]. The sequencing and annotation of complete prokaryotic and eukaryotic genomes has revealed the massive impact of TEs on genomic structure, evolution, and gene regulation [2-4]. Currently, most bioinformatics tools related to transposable elements are TE databases that collect and organize TE families in genomes (e.g. Repbase [5] and ISFinder [6] for eukaryotic and prokaryotic TEs, respectively); or detection methods (e.g. RepeatMasker [7], Censor [8], Repet [9]) that look for TE copies in sequences.

To our knowledge, the UCSC Genome Browser (genome.ucsc.edu/index.html) [10], ENSEMBL site (www.ensembl.org/index.html) [11], and DFAM database (www.dfam.org) [12] are the only Web browsers available that allow for the visualization and exploration of TE annotations. These browsers can display TEs with different resolutions, but they do not permit analyses and comparisons of individual TE families and superfamilies. Moreover, these browsers do not display similarities of TEs compared with their consensus sequences, which is essential for dating different generations of TEs. Previously, we developed VisualRepbase (www.girinst.org/downloads/software/) [13], a JAVA interface that browses for occurrences of TEs in annotated genomes based on their family name and their similarity to recognized consensus sequences, and allows the user to compare the age and the invasion origin of selected TEs. However, VisualRepbase suffers from a limited number of available genomes due to infrequent updates of the background database.

Furthermore, VisualRepbase, ENSEMBL, and the UCSC Genome Browsers do not show relationships between transposable elements and neighboring genes. Here, we describe a new stand-alone software named VisualTE that dynamically displays and analyzes occurrences of TE families within annotated genomes, based on TE similarity and size. VisualTE also exhibits TE relationships with neighboring gene features, as well as inter- and intra-chromosomal comparisons.

## Implementation and input data

VisualTE is written in the JAVA programming language (JAVA version 1.7 or later). The downloadable version can be installed and run on any operating system, including Windows, MacOS, and Linux.

VisualTE input data are divided into two categories: the annotation file (in Genbank and/or EMBL formats) and the repeat file. For the latter, VisualTE recognizes AB-BLAST [14], NCBI-BLAST [15], Censor [16], RepeatMasker [7], and Repet [9] formats. Moreover, a VisualTE format has been defined for the annotation and repeat files (see Additional file 1). A TE neighboring gene is defined as the closest annotated gene located upstream or downstream of a selected TE. VisualTE needs a file named ‘gene2go’ that can be downloaded from the NCBI website (ftp://ftp.ncbi.nlm.nih.gov/gene/DATA/) to analyze the Gene Ontology (GO) of these TE neighboring genes. VisualTE contains a TE superfamily information file that was extracted from the Repbase database (version 19.04) [5] and the ISFinder database (January 2014 version) [6]. Compared to VisualRepbase [13], VisualTE allows the input of any annotated sequence in the right format. For GO studies, the 148 generic GO categories (‘GenericEBI’) and the two first levels (‘TreeLevel1’ and ‘TreeLevel2’) of the GO hierarchical tree were extracted from the EBI (www.ebi.ac.uk/QuickGO/GMultiTerm\#tab=choose-terms) and Gene Ontology (ftp://ftp.geneontology.org/pub/go/ontology/go-basic.obo) websites, respectively. All of the information files from the Repbase, ISFinder, EBI, and GO websites will be regularly updated. The complete *Arabidopsis thaliana* genome [17] in Genbank format and reference Repbase families were downloaded from the NCBI (ftp://ftp.ncbi.nlm.nih.gov/genomes/Arabidopsis_thaliana/) and RepBase (www.girinst.org/repbase/) websites, respectively, for a case study. TE copies were identified using RepeatMasker [7] with default parameters.

## Results and discussion

**Figure 1:**
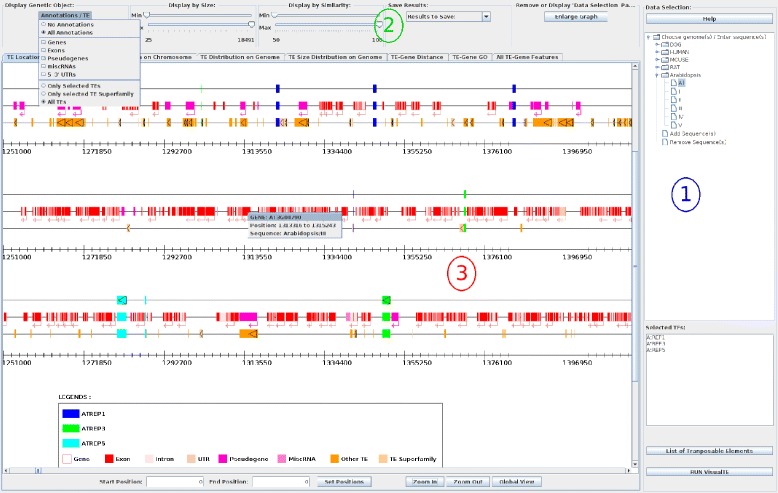
**Screenshot of the VisualTE interface.** The interface is divided into three areas: (i) the ‘Data Selection’ panel, (ii) the ‘Graphical Option’ selection header, and (iii) the ‘Graphical Panel’ (the ‘Location on Chromosome’ panel is visible). In the ‘Data Selection’ panel, the entire *Arabidopsis thaliana* genome and the Helitrons AtREP1, AtREP3 and AtREP5 are selected. The ‘Gene’ and the ‘Only selected TE Superfamily’ items are selected from the ‘Annotations/TE’ option. Each chromosome is represented by four lines. The three first lines correspond respectively to the selected TEs, the genes, and the TE superfamily lines. The last line is a graduated ruler line. The figure shows a popup menu displayed (gray rectangle) when the user clicks on a graphical item (here the gene). The two textfields (Start and End Position) and the ‘Set Positions’ button display the chromosome region between the two entered values. The ‘Zoom In’ (or ‘Zoom Out’) button increases (or decreases) by two fold the width of selected chromosomes. The ‘Global View’ button resizes the graphical view such that the largest chromosome is entirely included in the graphical panel width.

VisualTE is a graphical interface that reads, extracts, analyzes, and displays TE information from annotation and repeat data. The interface is composed of three distinct areas: ‘Data Selection’, ‘Graphical Option’, and ‘Graphical Panel’ (Figure 1). The last area, which constitutes the main part of the VisualTE tool, dynamically interacts with the ‘Option’ area through several buttons and functions. The AtREP1, AtREP3, AtREP5 families (194, 550, and 275 TE copies in *A. thaliana* genome, respectively) used in this work belong to the Helitron superfamily [18,19].

### Data selection area

This area is composed of a ‘Help’ button, a clickable genome tree, a textfield for entering a TE family name, a ‘List of Transposable Elements’ button, and the ‘RUN VisualTE’ button. Clicking on the ‘Help’ button opens a new interface window that explains all functions and buttons of the interface.

To use the VisualTE main interface the user starts by selecting one or several transposable element families (manually within the ‘Selected TEs’ area or from the ‘List of Transposable Elements’ button) with one or several genomic items within the ‘Data Selection’ area (Area 1 in Figure 1). The ‘Selected TEs’ textfield allows the user to enter the name of the desired TE family up to a maximum of 20 TE names (e.g AtREP1, AtREP3, AtREP5 in Figure 1). However, we recommend that the user limits this number to three TE names for better visualization. The ‘List of Transposable Elements’ button also opens a new interface window with the complete list of TE families generated from the input file (classified by organisms), and, therefore, allows for the selection of TE families of interest. The genome tree allows for the selection of particular chromosomes, as shown in Figure 1 for ‘All’ chromosomes of *A. thaliana*. Adding (or removing) new chromosome(s) to (or from) memory is accomplished by clicking on the ‘Add sequence(s)’ (or ‘Remove sequence(s)’) button in the interface. Finally, with at least one selected chromosome and at least one valid TE family name, the user runs the VisualTE program through the ‘RUN VisualTE’ button.

### Graphical option area

The ‘Option’ area includes (i) an ‘Enlarge Graph/Reduce Graph’ button that removes (or displays) the launch domain of the interface for better results visualization, and (ii) four options that dynamically interact with the ‘Graphical Panel area’ (Area 2 in Figure 1).

The ‘Annotation/TE’ menu displays genomic/TE annotations on chromosomes through the ‘Graphical Panel’ area. Genomic annotations include ‘Genes, Exons, PseudoGenes, miscRNA, and 5’- and 3‘-UTRs’, while TEs contain ‘Only selected TEs’, ‘Only selected TE Superfamily’, and ‘All TEs’ categories. An ‘All TEs’ item allows the user to display all TE copies within the selected chromosomes. The ‘Only selected TEs’ and ‘Only selected TE Superfamily’ choices do the same action for a specific TE family and superfamily, respectively. Each submenu independently displays (or removes) all genomic/TE annotations at the same time. The ‘Annotation/TE’ option is useful for examining TE copies according to their genetic environment.

The ‘Display by Size’ slider modifies all graphic panels and shows TEs that are respectively smaller and larger than the minimal and maximal values of the slider knob. By default, these values correspond to the smallest and the largest occurrences of the selected TEs, but can be dynamically changed by the user.

The ‘Display by Similarity’ slider exhibits and removes TEs that have respectively a lower and a larger similarity than the minimal and maximal values of the slider knob. The minimal and the maximal similarities (in comparison with the reference TE families) are set to 50% and 100% by default, respectively; but can be dynamically changed by the user. Since less divergent TE families are considered to be youngest ones, this slider can be used to estimate the evolutionary history of transposition in selected genomes.

The last item is a combo-list called ‘Save Results’. This list contains three saving options: the first two options save the whole graph or the visible part of the selected panel, while the last saving option writes out the TE occurrence list with their surrounding genes to a text file (as shown in the ‘All TE-Gene Features’ panel).

### Graphical panel area

Because TEs are involved in genome rearrangements and in the expression of various genes [20,21], this area contains seven graphical panels (Area 3 in Figure 1) that show the structural organization and functional relationships between TEs and their host genomes. The ‘Graphical Panel’ area dynamically displays the selected TEs, each with a specific color code.

#### TE location on chromosome

This panel, which was first described in VisualRepbase [13], draws selected TE copies as well as genomic annotation items on chromosomes. Figure 1 shows the AtREP1, AtREP3, and AtREP5 occurrences in blue, green, and light blue rectangles, respectively. By default, two lines representing the selected TE copies and a graduated ruler of the chromosome size are displayed. When ‘Only selected TE Superfamily’, ‘All TEs’ or/and genomic items (‘Genes’, ‘Exons’,...) are selected, new lines corresponding to the annotations or to TE copies are displayed between the two previous lines (as shown in Area 3 in Figure 1). Compared with the panel in VisualRepbase [13], this panel has an additional button (‘Set Positions’) and two additional textfields (linked to the ‘Set Positions’ button) that dynamically modify the graphical view. The textfields (Start and End Position) and the ‘Set Positions’ button display the chromosome region between the two entered values. In addition, when a user clicks on a graphical element, a menu with detailed information (nature of the genetic object and its location) is displayed (e.g. the detailed information shown for AT3G04790 gene in Figure 1, Area 3). Similar to VisualRepbase, the ‘Zoom In’ (or ‘Zoom Out’) button increases (or decreases) by two-fold the width of the selected chromosomes. The last button, ‘Global View’, resizes the graphical view such that the largest chromosome is entirely included in the graphical panel width. These three buttons also modify the display of the ‘Distribution on Chromosome’ panel.

#### TE distribution on chromosome

**Figure 2:**
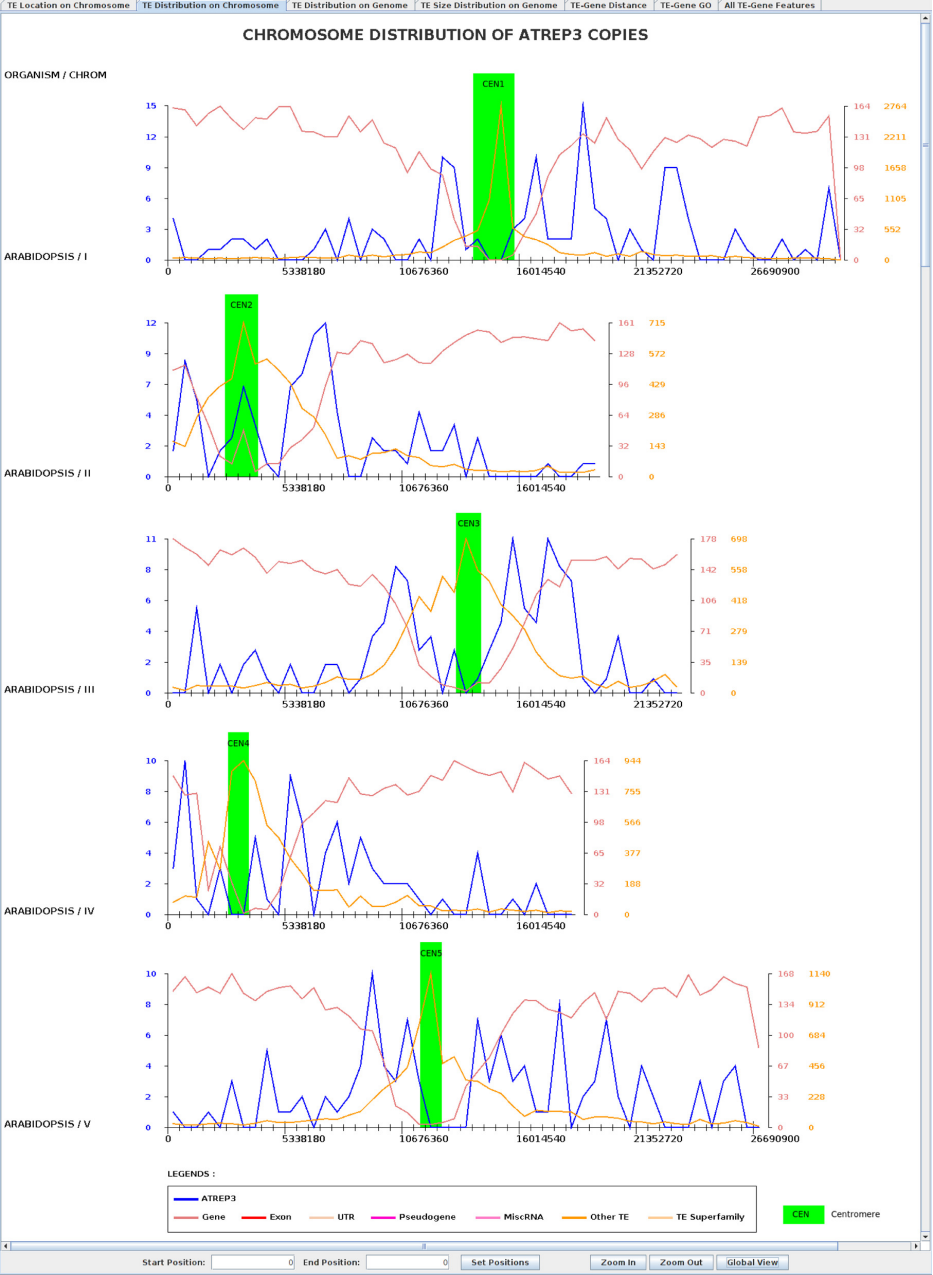
**AtREP3 distribution in** ***Arabidopsis thaliana*** **.** AtREP3, ‘All TEs,’ and gene distribution along the chromosomes are colored blue, orange, and light red, respectively. The same color code is used for the Y-scale. Centromeric regions from [22] are shown in green rectangles.

For each chromosome and each family, this panel draws the occurrence number of corresponding TEs along the chromosome (Figure 2). A new curve is dynamically displayed for any modification from the ‘Annotation/TEs’ menu. The same button and two textfields described in the ‘Location on Chromosome’ panel are also present in this panel. Comparative analysis of the TE/gene distribution in *A. thaliana* (Figure 2) shows that TEs are mainly located in centromeric and pericentromeric regions, while genes are underrepresented in these regions, as previously described [17,18]. TE overrepresentation in centromeric and pericentromeric regions result to their accumulation in these gene-pure, low-recombination regions, and in the effective removal of TEs inserted into gene-rich euchromatin regions.

#### TE distribution on genome

**Figure 3:**
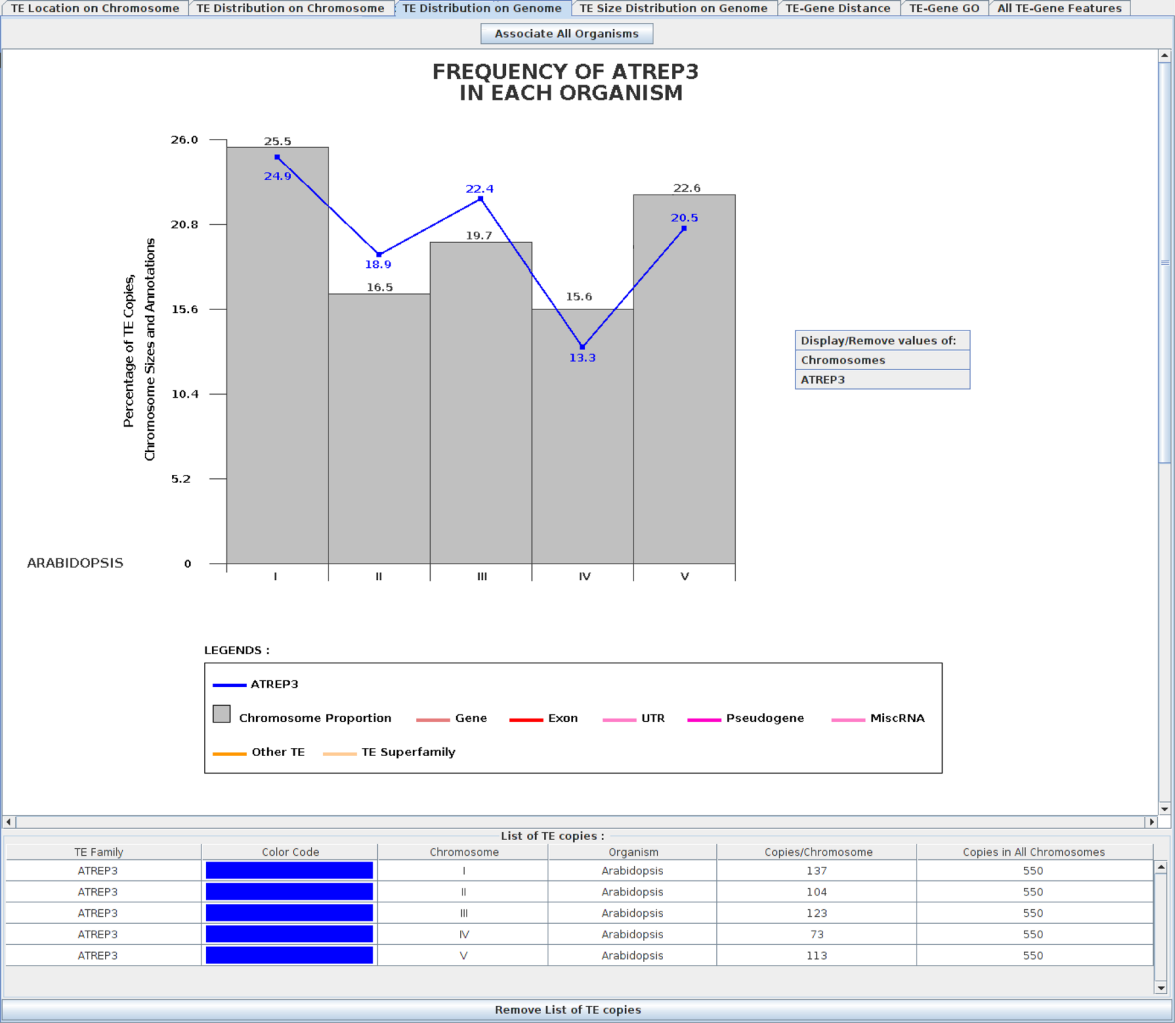
**AtREP3 frequency in** ***Arabidopsis thaliana*** **.** The top graphical part displays a histogram where each bar corresponds to the proportion of each chromosome size (grey bar). The blue curve is the AtREP3 distribution where each curve point corresponds to the percentage of AtREP3 copies in the *Arabidopsis* chromosomes. The ‘Associate Sequences’ button associates all histogram genomes into one histogram and vice versa (‘Dissociate Sequences’ button). The bottom graphical part displays a list of TEs with the number of copies on each chromosome and the total number of copies in *A. thaliana*. The ‘Remove List of TE copies’ button allows the TE list to be removed for better visualization of the histogram.

This panel is divided into two parts: (i) a graphical part that displays a histogram where each bar corresponds to the proportion of each chromosome length (compared against the overall genome) as well as a curve point corresponding to the percentage of each selected TE family (number of TE family copy over the total number of TE copies) for each selected chromosome; and (ii) a tabular part that summarizes the occurrence number of TE families for each chromosome. As an example, Figure 3 presents the AtREP3 TE family frequency in *A. thaliana*. AtREP3 copies (blue line) are not uniformly present on each chromosome, being overrepresented on chromosomes II and III (e.g. 19.7% (chromosome size proportion) versus 22.4% (AtREP3 frequency) for chromosome III). The overrepresentation of AtREP3 copies in *Arabidopsis* chromosomes may correspond to new genomic functions, as has been reported with L1 families in mammals [23,24]. Therefore, this panel, as with the previous one, may help to identify strong insertion biases towards specific TEs and chromosomes, and potentially identify new functions associated with TEs.

#### TE size distribution on genome

**Figure 4:**
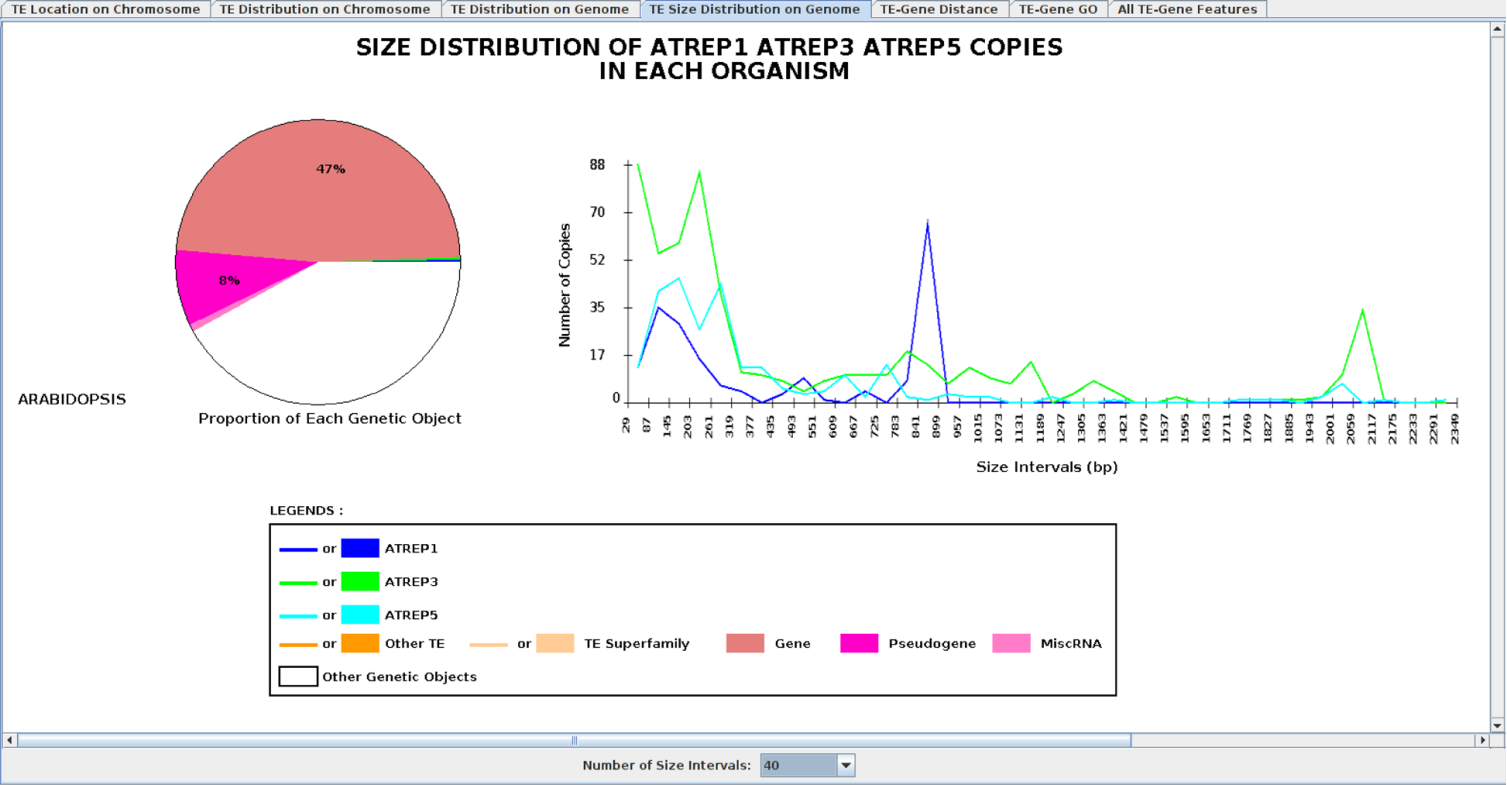
**Screenshot of the ‘Size Distribution’ panel.** Proportions of each genetic object and the size distributions of AtREP1, AtREP3, and AtREP5 in *Arabidopsis thaliana* are shown. The ‘All TEs’ and ‘Genes’ items were selected in the ‘Annotation/TE’ menu. The scale size of the curve can be changed anytime through the ‘Display by Size’ slider and the ‘Number of Intervals’ button. The number of size intervals is set to 40.

This panel draws two graphs: a pie chart size distribution of genetic items and a distribution size curve for each selected TE from each selected genome. The pie chart distribution of genetic items shows, for each selected TE family and each selected item from the ‘Annotation/TEs’ menu, the total size proportion of those items on the genome. In Figure 4, ‘All TEs’, ‘Genes’, ‘Pseudo’, and ‘miscRNA’ are selected and their total size proportion in the *Arabidopsis* genome is displayed. This proportion (here 47% and 8% for genes and pseudogenes, respectively) may be useful for understanding the overall content of each genetic object. Variations in TE sizes shown in the distribution size curve may reflect the evolution of the TE family. In fact, a high number of identical TE copies of similar size indicates young or recent TE copy insertions. Contrastingly, an old TE family exhibits many mutations (insertions/deletions) leading to a high heterogeneity of TE sizes, as shown for the families of AtREP (AtREP1, AtREP3, and AtREP5) copies (Figure 4). Indeed, the AtREP1 and AtREP3 curves present two main peaks (∼850 bp and ∼2100 bp, respectively) corresponding to the reference consensus sizes; and TEs of smaller sizes (<300 bp) are observed for AtREP1, AtREP3, and AtREP5, which most likely resulted from fragmented TE identification.

#### TE-gene distance

**Figure 5:**
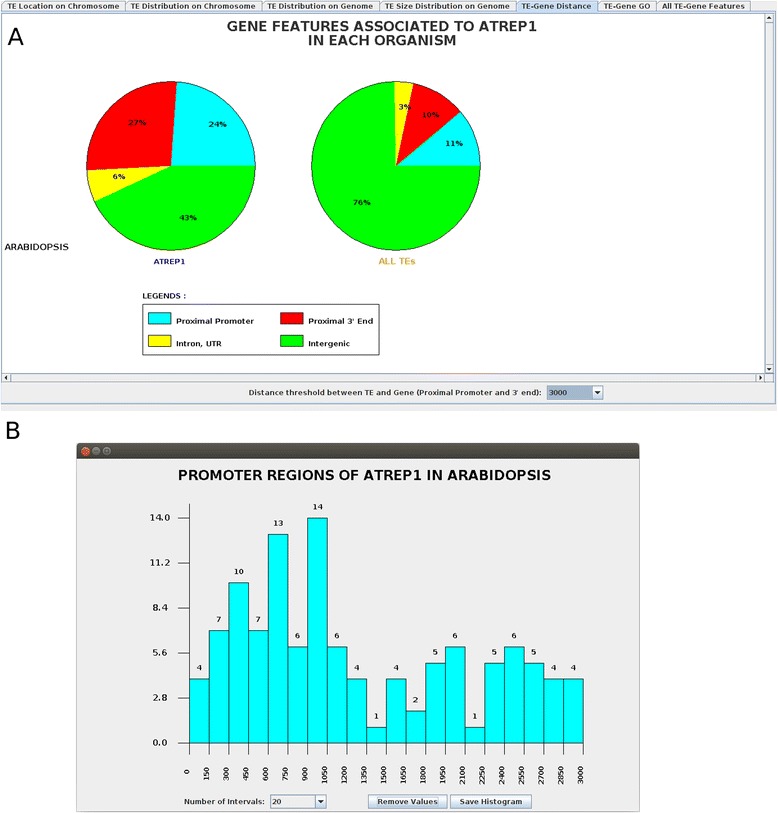
**Screenshot of the ‘Gene Distance Frequency’ panel displaying the AtREP1 and the helitron superfamily distribution.** **A)** This panel shows a pie chart of the AtREP1 copy distribution relative to the following genetic regions: ‘Proximal Promoter,’ ‘Proximal 3’ end,’ ‘Intron/UTRs,’ and ‘Intergenic.’ **B)** When a user clicks on a pie chart (here, on the blue slice representing ‘Proximal Promoter’), a new interface panel opens and shows a histogram corresponding to the AtREP1 copy distribution within the clicked region. The number of intervals is set to 20 and a ‘Save Histogram’ button is used to save the histogram image. A ‘Display Values’ (or ‘Remove Values’) button displays (or removes) the number of observations within the histogram.

For each TE family in each organism, this panel shows a distribution pie chart of the following gene feature regions: ‘Proximal Promoter’, ‘Proximal 3’ End’, ‘Exon/Intron/UTRs’, and ‘Intergenic’. By default, a TE copy belongs in the ‘Proximal Promoter’ or ‘Proximal 3’ End’ category, if the distance between the TE copy and the corresponding neighboring gene is equal to or less than 3,000 bp. This distance value can be changed via a combo-list button. When a user clicks on a pie chart colored segment, a new interface window is opened that shows a histogram corresponding to the TE copy distribution of the clicked gene feature region. The selection of TE items in the ‘Annotation/TE’ menu displays a new pie chart for each genome. Figure 5 illustrates the distribution of the AtREP1 family in the previously defined regions (A in Figure 5), and the histogram distribution of AtREP1 in the ‘Proximal Promoter’ region (B in Figure 5). Therefore, AtREP1 is mainly located in intergenic regions (37%, including centromeric regions) and near genes (‘Proximal Promoter’ 27% or ‘Proximal 3’ End’ 30%), while overall TEs are preferentially inserted within intergenic regions (71%). Similar findings were observed for Helitron copies in *Aspergillus nidulans* and *Drosophila melanogaster* [25,26]. Moreover, the histogram at the bottom of Figure 5B demonstrates that the AtREP1 family is preferentially inserted near the start position of the gene, suggesting a possible role of AtREP1 in gene regulation. Altogether, this panel allows the user to examine the relative location (distance and orientation) of TE copies and genes, and, therefore, may indicate possible roles of TEs in gene regulation.

#### TE-gene gene ontology

**Figure 6:**
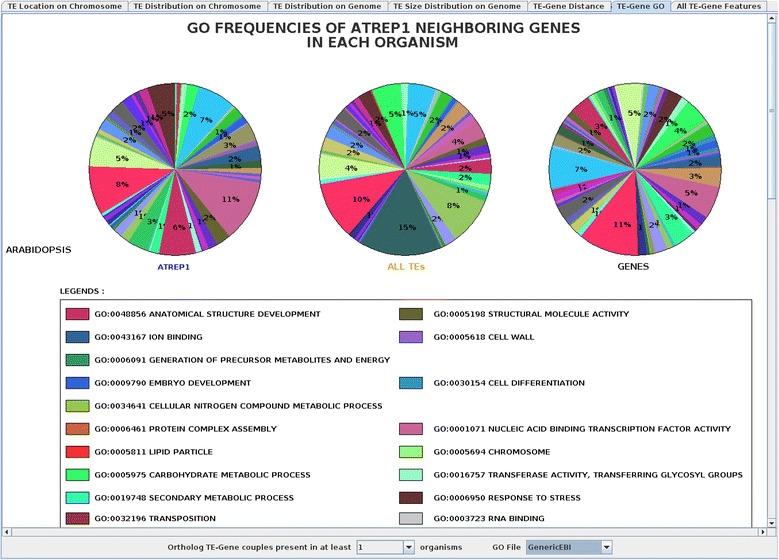
**Pie chart distribution of AtREP1, the entire helitron superfamily, and genes from the ‘Gene Ontology’ panel for** ***Arabidopsis thaliana*** **.** Here, the orthologous TE-gene couples should be present in at least one organism and the GenericEBI file is selected from the ‘GO File’ combo-list. Each color corresponds to one GO family and proportions higher than 1% are displayed.

This panel shows pie charts of gene ontology distributions related to neighboring genes (next downstream and next upstream genes) for the selected TE families and TE superfamilies, as well as from overall genes. Pie charts are dynamically changed with any modification in the ‘Annotation/TE’ selection. A combo-list allows for the selection of TE-gene couples that are present in at least *X* number of organisms, to allow for comparative analyses between several organisms. Because the same combo-list is also defined in the ‘All TE-Gene Features’ panel, any change of the *X* value results in the same modification in the ‘All TE-Gene Features’ panel as well. For user convenience, three different Gene Ontology (GO) lists (see Input Data) can be selected through a ‘GO File’ combo-list. Figure 6 shows the GO distribution of the *Arabidopsis thaliana* genes, and for the closest neighboring genes, to the AtREP1 copies. This figure clearly highlights an overrepresentation of the AtREP1 insertions near genes involved in GO categories such as ‘Anatomical Structure Development’ (GO:0048856), ‘Transposition’ (GO:0032196), and ‘Nucleic Acid Biding Transcription Factor Activity’ (GO:0001071). This panel may prove useful in examining functional relationships between TEs and neighboring genes.

#### All TE-gene features

This panel summarizes all of the results from the six previous panels into a table for download and further analysis (see Additional file 2). At least two lines for each TE copy correspond to the upstream and downstream genes closest to each TE. A third line is displayed, if the TE copy is inserted within a gene. For each TE, each line contains the TE location and orientation along the chromosomes, the superfamily name, and the similarity (compared with the consensus), as well as the name, the positions, and the orientation of gene, the distance between the neighboring gene and the TE, and the GO family. The last column (‘Ortholog’) represents the TE-gene couple *X* values as defined before. Moreover, when many genomes are selected, this panel allows a user to identify the TE copies that are conserved (or inserted) close to the same orthologous genes.

## Conclusions

VisualTE is a stand-alone JAVA interface that allows users to analyze and visualize the size, the intra-chromosomal and inter-chromosomal copy distribution, and the genetic distance distribution of TE copies. Indeed, the ‘TE-Gene Distance’ graph which examines the relative location between the TE copies and genes, may indicate a role of TE in gene regulation. VisualTE should help researchers identify strong insertion biases toward specific TEs and chromosomes, leading to the discovery of TE functions. Moreover, it easily allows a user to perform comparative analyses with these TEs and any other genetic objects, including genes, exons, UTRs, pseudogenes, and miscRNAs. VisualTE can also exhibit the conserved couple TE-‘orthologous neighboring genes’ with their GOs in selected organisms, which could prove useful for examining functional relationships between TEs and neighboring genes. In summary, this graphical interface makes TE diversification studies possible in a single analysis, and thus might provide clues for understanding TE dynamics at the whole-genome scale.

## Availability and requirements

**Project Name:** VisualTE**Project home page:**http://lcb.cnrs-mrs.fr/spip.php?article867**Operating system(s):** Platform independent**Programming language:** JAVA**Licence:** Creative Common v3**Any restrictions to use by non-academics:** No.

## Additional files

Additional file 1
**Supplementary Data 1.**

Additional file 2
**Supplementary Data 2.**
