# Supplementary material for: VisualTE: a graphical interface for transposable element analysis at the genomic scale
Source: BMC Genomics. 2015 Feb 27;16(1):139. doi: 10.1186/s12864-015-1351-5 (PMC4367877; doi:10.1186/s12864-015-1351-5)
Supplement: Additional file 1 — Supplementary Data 1. [file 12864_2015_1351_MOESM1_ESM.pdf]

Supplemantary Data 1

```
Annotation file:
15528545
85401 98186 GENE ORC4 d 76 GO:0003810 GO:0005634
85401 85445 UTR ORC4 d 5'
85446 86701 EXON ORC4 d 1
85701 89890 INTRON ORC4 d 1
89891 90017 EXON ORC4 d 2
...
```

The first line corresponds to the overall sequence size. The other lines are genetic object annotations, where columns are: the start and end locations, genetic object (GENE, misc RNA, PSEUDO, UTR, INTRON EXON), genetic object name, orientation, and associated gene ontology for the first five columns and the last column, respectively. The sixth column depends on the genetic object: it corresponds to the position order along the chromosome for a gene, pseudogene, and miscRNA objects (in this example, the ORC4 gene is the 76th object within the chromosome), or the symbol 5' or 3' for UTRs. All columns are separated by tab characters, except for the list of GO entries in the last columns, which are separated by space characters.

```
Repeat file:
79007 79048 TE ATHPOGO c 93
79976 80047 TE ATENSPM5 d 83
...
```

Where columns are: the genetic object's start and end locations, the genetic object (TE here), the genetic object's name, its orientation, and the percentage of similarity with its consensus sequence. All columns are separated by tab characters.
