# Supplementary material for: VisualTE: a graphical interface for transposable element analysis at the genomic scale
Source: BMC Genomics. 2015 Feb 27;16(1):139. doi: 10.1186/s12864-015-1351-5 (PMC4367877; doi:10.1186/s12864-015-1351-5)
Supplement: Additional file 2 — Supplementary Data 2. [file 12864_2015_1351_MOESM2_ESM.pdf]

TE Location on Chromosome | TE Distribution on Chromosome | TE Distribution on Genome | TE Size Distribution on Genome | TE-Gene Distance | TE-Gene GO | **All TE-Gene Features**

Genome	Chrom	TE Family	Color	TE Start	TE End	Direction	Similarity	Superfamily	Gene Dista...	Gene Positi...	Gene Start	Gene End	Gene	Gene Direc...	GeneOntolo...	Ortholog
Arabidopsis	I	ATREP1		9918887	9918986	d	71	HELITRON	5050	5'	9911898	9913837	AT1G28310	c	GO:000367...	1
Arabidopsis	I	ATREP1		9918887	9918986	d	71	HELITRON	1521	3'	9920507	9924488	DEG15	c	GO:000367...	1
Arabidopsis	I	ATREP1		11782860	11783036	c	70	HELITRON	5303	5'	11776765	11777557	AT1G32570	d	GO:000367...	1
Arabidopsis	I	ATREP1		11782860	11783036	c	70	HELITRON	1021	3'	11784057	11785637	AT1G32580	d	GO:000367...	1
Arabidopsis	I	ATREP1		13465359	13465489	c	63	HELITRON	9028	5'	13454496	13456331	AT1G36060	c	GO:000367...	1
Arabidopsis	I	ATREP1		13465359	13465489	c	63	HELITRON	1422	3'	13466911	13470936	AT1G36070	c	GO:000367...	1
Arabidopsis	I	ATREP1		17707257	17707384	c	68	HELITRON	797	5'	17704179	17706460	MYB112	c	GO:000573...	1
Arabidopsis	I	ATREP1		17707257	17707384	c	68	HELITRON	4107	3'	17711491	17712660	AT1G48010	c	GO:000367...	1
Arabidopsis	I	ATREP1		18281609	18281759	c	64	HELITRON	631	5'	18279600	18280978	AT1G49390	d	GO:000002...	1
Arabidopsis	I	ATREP1		18281609	18281759	c	64	HELITRON	1273	3'	18283032	18284202	EMB1129	d	GO:000573...	1
Arabidopsis	I	ATREP3		13465381	13465676	c	68	HELITRON	9050	5'	13454496	13456331	AT1G36060	c	GO:000367...	1
Arabidopsis	I	ATREP3		13465381	13465676	c	68	HELITRON	1235	3'	13466911	13470936	AT1G36070	c	GO:000367...	1
Arabidopsis	I	ATREP3		16454400	16454520	d	70	HELITRON	7514	5'	16443685	16446886	AT1G43650	c	GO:000370...	1
Arabidopsis	I	ATREP3		16454400	16454520	d	70	HELITRON	6552	3'	16461072	16461734	AT1G43666	c	GO:000367...	1
Arabidopsis	I	ATREP3		16998958	16999212	c	71	HELITRON	3663	5'	16995113	16995295	AT1G44941	c	GO:000367...	1
Arabidopsis	I	ATREP3		16998958	16999212	c	71	HELITRON	326	3'	16999538	17001729	AT1G44960	d	GO:000367...	1
Arabidopsis	I	ATREP3		16999110	16999225	c	65	HELITRON	3815	5'	16995113	16995295	AT1G44941	c	GO:000367...	1
Arabidopsis	I	ATREP3		16999110	16999225	c	65	HELITRON	313	3'	16999538	17001729	AT1G44960	d	GO:000367...	1
Arabidopsis	I	ATREP3		18281336	18281470	c	66	HELITRON	358	5'	18279600	18280978	AT1G49390	d	GO:000002...	1
Arabidopsis	I	ATREP3		18281336	18281470	c	66	HELITRON	1562	3'	18283032	18284202	EMB1129	d	GO:000573...	1
Arabidopsis	I	ATREP3		18905797	18905959	d	56	HELITRON	593	5'	18902930	18905204	AT1G50990	d	GO:000367...	1
Arabidopsis	I	ATREP3		18905797	18905959	d	56	HELITRON	3965	3'	18909924	18910142	AT1G51000	d	GO:0005886	1
Arabidopsis	I	ATREP3		21069812	21069956	c	66	HELITRON	3959	5'	21064279	21065853	AT1G56260	c	GO:000551...	1
Arabidopsis	I	ATREP3		21069812	21069956	c	66	HELITRON	1061	3'	21071017	21071650	AT1G56270	c	GO:000072...	1
Arabidopsis	I	ATREP3		22782225	22782417	d	70	HELITRON	837	5'	22780733	22781388	AT1G61688	c	GO:000039...	1
Arabidopsis	I	ATREP3		22782225	22782417	d	70	HELITRON	190	3'	22782607	22786859	AT1G61690	c	GO:000950...	1
Arabidopsis	I	ATREP5		822388	822528	c	59	HELITRON	1071	5'	819662	821317	AT1G03340	d	GO:000403...	1
Arabidopsis	I	ATREP5		822388	822528	c	59	HELITRON	110	3'	822638	824393	AT1G03350	c	GO:000535...	1
Arabidopsis	I	ATREP5		10920945	10921249	c	71	HELITRON	268	5'	10918278	10920677	AT1G30760	d	GO:000367...	1
Arabidopsis	I	ATREP5		10920945	10921249	c	71	HELITRON	2670	3'	10923919	10926561	AT1G30780	c	GO:000573...	1
Arabidopsis	I	ATREP5		11408057	11408563	c	61	HELITRON	1798	5'	11399607	11406259	AFH14	c	GO:000002...	1
Arabidopsis	I	ATREP5		11408057	11408563	c	61	HELITRON	2312	3'	11410875	11412204	ACBP6	c	GO:000377...	1
Arabidopsis	I	ATREP5		11452926	11453079	c	71	HELITRON	941	5'	11450460	11451985	NIP3;1	d	GO:000563...	1
Arabidopsis	I	ATREP5		11452926	11453079	c	71	HELITRON	4047	3'	11457126	11461825	AT1G31910	d	GO:000681...	1
Arabidopsis	I	ATREP5		12039671	12039823	d	63	HELITRON	27147	5'	12010901	12012524	AT1G33120	d	GO:000521...	1
Arabidopsis	I	ATREP5		12039671	12039823	d	63	HELITRON	6818	3'	12046641	12050066	AT1G33230	d	GO:000455...	1
Arabidopsis	I	ATREP5		13465796	13465965	c	67	HELITRON	9465	5'	13454496	13456331	AT1G36060	c	GO:000367...	1
Arabidopsis	I	ATREP5		13465796	13465965	c	67	HELITRON	946	3'	13466911	13470936	AT1G36070	c	GO:000367...	1

Ortholog TE-Gene couples present in at least 1 **organisms**
